# Psychological Effects of Social Isolation Due to Quarantine in Chile: An Exploratory Study

**DOI:** 10.3389/fpsyt.2020.591142

**Published:** 2020-11-17

**Authors:** Paula Dagnino, Verónica Anguita, Katherine Escobar, Sofía Cifuentes

**Affiliations:** ^1^Faculty of Psychology, Universidad Alberto Hurtado, Santiago, Chile; ^2^Center for the Research on Psychotherapy—CIPSI, Santiago, Chile; ^3^Millennium Institute for Research on Depression and Personality—MIDAP, Santiago, Chile; ^4^Ethics Committee of Research With Human Beings, Universidad Alberto Hurtado, Santiago, Chile; ^5^Bioethics Department, Faculty of Medicine, Universidad de Chile, Santiago, Chile

**Keywords:** psychological impact, COVID-19, psychological support, tele-psychotherapy, vulnerability factors

## Abstract

As we all know, COVID-19 has impacted the entire world. Quarantine disrupts people's lives, with high levels of stress and negative psychological impacts. Studies carried out mostly in the Far East, Europe, or the United States have started to provide evidence on survivors, frontline healthcare workers, and parents. The present study is the first survey to be carried out in Latin America (in Santiago, the capital of Chile). It aims to (a) explore the perceived psychological impact and future concerns; (b) evaluate vulnerability factors; (c) describe the perceived psychological impacts on participants whose psychological help and actual online psychotherapy was interrupted; and (d) explore the future need for psychological help. *Procedure:* An online survey was carried out (the first 2 weeks of lockdown in Santiago), which included sociodemographic data, perceived psychological impact, future concerns, and questions about psychological support. *Participants:* A total of 3,919 subjects answered, mostly women (80%). *Results:* The main perceived psychological impacts were concern (67%) and anxiety (60%). *Future concerns were*: general health (55.3%), employment (53.1%), and finances (49.8%). Younger participants had a greater perceived psychological impact (*p*'s < 0.01) and concerns about employment, finances, mental health, stigma, and general health (*p*'s < 0.001). Women reported more perceived psychological impact than men (*p*'s < 0.05). Men reported mainly boredom (χ^2^ = 11.82, gl = 1, *p* < 0.001). Dependent employees experienced more boredom, anxiety, distress, sleep problems, an inability to relax, and a lack of concentration than the self-employed (p's < 0.05). While the latter reported future concerns about employment and finances (*p*'s < 0.001), dependent employees reported them on their general and mental health (*p*'s < 0.001). Regarding psychological support, 22% of participants were receiving it before lockdown. They showed more perceived psychological impact than those who were not (*p*'s < 0.01), and 7% of them had online psychotherapy, reporting excellent (32.1%) or odd but working (65.2%) results. Finally, of the total sample, almost half of the participants (43.8%) felt they would need emotional support after this pandemic, and these are the ones that also showed higher perceived psychological impact (*p*'s < 0.001). This study confirms the presence of perceived negative emotional impact and concerns about the future. Also, there are vulnerable groups, such as women, younger people, the self-employed, and people with psychological processes that were interrupted.

## Introduction

There have been several pandemics in human history (e.g., Spanish Flu, 1918–1919; Asian Flu, 1957–1958; A1NH1 2009–2010). The current pandemic, COVID-19, has impacted the entire world, starting in China in December 2019. At the time of writing the manuscript, COVID-19 was affecting 213 countries and territories around the world, with almost 16,575,090 cases and 654,623 deaths (1). In Chile, the first case of COVID was a 33-year-old man who had to be hospitalized on 3 March. The first death took place on 21 March.

This study was carried out in Chile between 23 March and 15 April 2020. At the end of the survey, 95 people had died out of 8,273 confirmed cases (2). Artificial respirators and ICUs were at ≤ 20% occupancy (3).

In the history of pandemics, one of the viruses with a worldwide impact was H1N1, which had an infection rate of ~1.6 infected by one subject with the virus, and its symptomatology was quickly evident (4, 5). COVID-19 has a higher level of contagion, estimated at 4.08 (5), and it also presents characteristics that are mutating, such as the symptoms and presence of the virus, even in asymptomatic conditions (4–6). These cause high levels of anxiety and fear about the future (7, 8). Countries around the world have established more or less strict quarantine measures. It is believed that confinement, such as social isolation, helps to prevent the spread of the virus (9).

Quarantine or social isolation disrupts people's jobs and lives immensely, and hence it may have important implications for their health and well-being (7, 8). These necessary measures lead the general population to a high level of stress and psychological problems, producing uncertainty, fear of contagion, and illness in themselves and their loved ones, and a fear of financial loss (7, 10, 11). Separation from loved ones, loss of freedom, losing direct social contact, employment, recreation, privileges, boredom (12), and uncertainty over the disease's status, on occasion, create dramatic effects that are among the significant stressors that will undoubtedly contribute to widespread emotional distress (13).

Indeed, the well-being implications of quarantine were evident in previous outbreaks such as SARS or MERS. After quarantine, hospital staff showed more acute stress disorders (13) and post-traumatic symptoms, even 3 years later (14). Among the general population, anger and anxiety were predominant, mainly because of economic concerns several months later (13), with an increase in the number of suicides (15, 16).

The studies that have been done so far on the COVID-19 pandemic, and especially on the effects of quarantine, have shown a high negative psychological impact. These studies were conducted mostly in the Far East, Europe, or the United States, and they have started to provide showing adverse emotional effects such as increased stress, depression, anxiety, sleeping difficulties, post-traumatic stress, anger, boredom, stigma, substance use, and loneliness (17–28). Moreover, some studies have compared negative emotions before lockdown and during the COVID-19 outbreak. The results have shown that there has been an increase in negative emotions during lockdowns, such as anxiety and depression (7, 29), and a decrease in life satisfaction (30). After 1 month of confinement, Zhang et al. (22) found that those who stopped working reported worse mental health and more distress. Because of these long-lasting effects, it is extremely relevant to enquire about the actual psychological impact and future concerns during this particular pandemic quarantine in other regions, such as South America.

From all of the studies, it is evident that pandemics such as this one, and its concomitant lockdowns, have a massive impact on people, especially on their mental health, which includes different feelings about it and future concerns. Identifying these is very important in terms of taking measures to prevent or treat the psychological impact. Few studies have evaluated these issues among the general population (25, 31), mainly targeting specific people such as health professionals [e.g., (4, 32–34)], COVID-19 survivors (33), or specific age groups (35).

From COVID-19 studies, particular vulnerability factors have been identified. As such, they increase the presence of a negative emotional impact due to quarantine. Some of these are gender, educational level, and age [e.g., (28, 36)]. Findings on gender suggest that women are more vulnerable to stress than their male counterparts (4, 37), increasing the possibility of developing post-traumatic disorders afterwards (19, 31). On the other hand, people with a higher level of education tend to have more distress, probably because of a high level of self-awareness about their health (19, 37). Concerning age, individuals between 18 and 30 years of age, or above 60, presented the highest levels of emotional distress (19). Younger people, such as college students, showed that they were experiencing anxiety during COVID-19 (4, 35). Other studies found that one of the vulnerability factors for screening anxiety or depression was a younger age (31, 36). Some studies in Italy have related the impact of quarantine to personality traits such as negative affect and attachment, finding that detachment and negative affect were related to depression, anxiety, and stress (25).

Besides vulnerability factors, concerns about the present and future are among the main issues that lead to high stress during lockdown. As is expected for infectious disease, the main concern is about becoming sick or that a family member will (7). However, there have also been high economic consequences related to other pandemic conditions (38). In the SARS outbreak in Canada and the United States, concern about financial loss meant that people did not comply with quarantine or evacuation (37, 39–41). Also, months after SARS struck in China, the fear of income reduction was among the highest vulnerability factors for psychological disorders (39). Specifically, with the COVID-19 pandemic, college students have been worried about the economic influences of the epidemic, which are related to the high levels of anxiety (24, 35). Therefore, not only are the concerns about health issues relevant, but economic or financial matters will also be highly prevalent.

However, there is another part of the population that has gone unnoticed, namely, those who were having psychological help before lockdown. Few studies have focused on this type of person, concentrating instead on psychiatric patients or inpatients [e.g., (42)], showing that quarantine exacerbates existing mental health disorders (43–45), or asking about psychological support or psychotherapy as one of the areas but not relating it to psychological distress or symptoms (25). Diagnosed before the pandemic, mental health symptoms are associated with anxious and irritable symptoms 4–6 months after quarantine (46, 47). However, in the area of mental health, not only must people with severe mental disorders be taken into account, there are also people who were having psychological outpatient help before lockdown (48). These people have faced not only the impact of social isolation and quarantine, but also without this support. For this reason, they need to be evaluated, as they are likely to have a different or more intense perceived psychological impact.

We know that there has been an increase in the development of online psychotherapy (i.e., providing mental healthcare remotely, using telecommunications such as telephone or video conferencing tools), which has been introduced suddenly and expanded significantly to serve patients at treatment or in actual need of treatment (49). Many discussions of clinicians on organizations and some qualitative studies (50) have emerged with the intention of evaluating the impact of this new approach on therapists and their settings, and yet, no review has asked patients how they have experienced this change. This is an essential issue, since other pandemics had shown that mental health support and follow-up should be provided even 6 months after release from isolation for those individuals with or without a prior vulnerable mental health status (51). How patients evaluate the effectiveness of this new approach will be relevant to installing it as a modality to be performed in the future, in both online psychotherapy and online psychiatry.

The COVID-19 epidemic has caused a parallel epidemic of fear, anxiety, and depression worldwide, along with concerns about the future. This study is the first to evaluate the perceived psychological impact on a South American country such as Chile. The objectives are to (a) survey the general public to understand better their levels of psychological effect and future concerns; (b) identify relationships between vulnerability factors (age, gender, and occupation), perceived psychological impact, and future interests; (c) describe the perceived psychological effects on survey participants who had processes interrupted because of this pandemic, and evaluate online psychotherapy; and (d) explore the future need for psychological help. It is hypothesized that there will be perceived psychological impacts, such as anxiety and depression. Also, future concerns will appear mainly related to overall health and economic issues. Furthermore, vulnerability factors such as being a woman, younger, and self-employed may reveal differences. Finally, it is expected that those who had their processes interrupted will be more emotionally affected and that virtuality may help them. Finally, it is expected that there will be a significant percentage of people who think they will need psychological help in the future.

## Materials and Methods

### Participants

The sample comprised 3,919 participants, living in Santiago (the capital of Chile) (80% women). Participants' age ranges were created based on a quantile cutoff criterion, using the 20, 40, 60, and 80 quantiles. Accordingly, 20.0% of participants ranged from 18 to 29 years of age, 20.3% were between 30 and 38 years of age, 21.5% were between 39 and 46 years of age, 18.8% were between 47 and 55 years of age, and, finally, 19.37% participants were between 55 and 89 years of age. For the sample, only participants over 18 years of age were considered. Forty-six percent of the participants reported being employed workers, while 26% reported being self-employed.

### Procedure

In order to fulfill the objectives, an online survey was carried out (disseminated through personal and social networks) from 23 March to 15 April 2020 (23 days). This coincided with the first case of COVID-19 in Chile and the government's subsequent decision to keep the pandemic under control through a lockdown of Santiago.

This study was reviewed and approved by the Institutional Review Board of the Universidad Diego Portales (N°006-2020), which conformed to the principles embodied in the Declaration of Helsinki. All respondents provided informed consent.

### Measures

#### Survey Development

As a result of the need to screen several psychological symptoms that could appear during the COVID-19 pandemic, the survey items were developed with a focus on previous surveys on the psychological effects of SARS, Ebola and influenza outbreaks, and actual studies on COVID-19 (see *Introduction*). Specifically, we focused on Brooks et al.'s (52) revision, since it provided very thorough evidence on the psychological impact of quarantine. Brooks et al. (52) reviewed 903 studies on the prevalence of psychological symptoms, and they found that the most prevalent were insomnia, irritability, fear, stress, depression, concern, anxiety, and fear. Other input was Taylor's book (17), with the most reported psychological impact in almost all pandemics being anxiety, concern, fear, stress, uncertainty, irritability, and depression.

New dimensions appeared in Zhang et al. (22), which considered working conditions to be a vulnerability factor for psychological distress. Other authors [e.g., (52, 53)] mentioned the effect on financial loss of quarantine and therefore economic concerns as a stressor. With this input, the authors decided to separate the psychological impact from concern, mainly because quarantine was just starting in Chile and the future was therefore a big issue.

On the other hand, as three of the four authors are clinical psychologists, the question was quickly raised about how patients who were having help were managing to cope without it, and virtuality was also increasingly being used in Chile at the time, so there was a need to understand the subjective experience of patients of this new helping tool.

Therefore, the survey consisted of 16 questions, which evaluated several areas: (a) sociodemographic data (gender, age, education, and occupation); (b) the perceived psychological impact of quarantine; (c) future concerns; and (d) psychological support (pre-quarantine support, actual support, and future support needs).

Regarding the actual perceived psychological impact, respondents had to select one or more of the following perceived impacts: boredom, distress, anxiety, lack of concentration, frustration, inability to relax, restlessness, irritability, fear, loss of control, loss of freedom, concern, sleep problems, feeling trapped, and loneliness. On future concerns, respondents also had to select one or more of the following concerns: policy, school, economic issues, work, mental health, overall health, and stigma.

On psychological support, respondents were asked if they were receiving psychological help before quarantine [0 = no; 1 = yes], and those who answered affirmatively to this question had to answer the next questions as a sub-sample. First, if they had psychological support, 16 questions had to be answered: how long they had been receiving help when quarantine started (a few sessions, more than 6, or more than 12). They were asked about why they had started psychotherapy, and they could choose more than one alternative from depression, anxiety, psychosis, cognitive and learning problems, personality problems, eating disorders, physical problems, addictions, trauma abuse, grief, self-esteem, interpersonal relations, life and well-being, work, or study. They were also asked if they had psychological help online [0 = no; 1 = yes], and how they rated this new tool (excellent, odd but it works, it generates disgust, or no good).

Another aspect related to psychological support was to ask about the future need for psychological help after lockdown [0 = no; 1 = yes]. For this question, all survey respondents had to answer.

### Data Analysis

Given the exploratory nature of the study, the data analysis had two pivotal moments. The first moment consisted of the estimation of descriptive statistics for each of the variables of interest. Most of the study variables were measured as categorical variables, so their frequencies and total percentages were studied. In a second moment, bivariate relations between the variables of interest were calculated. When two categorical variables were associated, a chi-squared statistic was used to determine a statistically significant relationship, and when a categorical variable was related to a quantitative one, the Student *t* statistic was used to determine statistically significant differences. All statistical analyses were carried out using R v4.0.0 software (54).

## Results

### Perceived Psychological Impact and Future Concern

Table 1 shows the percentage of data of the different perceived psychological impacts reported by the study participants. It can be seen that the most frequently reported feeling was concern, with 67% of people reporting it. Next, the second most frequently reported perceived impact was anxiety, with 60% of the sample reporting feeling it during quarantine. On the other hand, feelings of loneliness were the second least reported, namely, 16% of the sample, and a feeling of loss of control was the least reported perceived impact, with only 9.5% of participants reporting feeling it.

**Table 1:** Number and percentage of reports of the current perceived impact and future concern.

	**Does not report**		**Reports**	
	***f***	**%**	***f***	**%**
**Current impact**				
Fear	2,648	67.6	1,271	32.4
Concern	1,288	32.9	2,631	67.1
Frustration	2,898	73.9	1,021	26.1
Boredom	2,556	65.2	1,363	34.8
Anxiety	1,556	39.7	2,363	60.3
Distress	2,324	59.3	1,595	40.7
Feeling trapped	2,991	76.3	928	23.7
Loss of control	3,546	90.5	373	9.5
Loneliness	3,283	83.8	636	16.2
Sleep problems	2,329	59.4	1,590	40.6
Inability to relax	2,962	75.6	957	24.4
Loss of freedom	2,716	69.3	1,203	30.7
Lack of concentration	2,476	63.2	1,443	36.8
Irritability	2,370	60.5	1,549	39.5
Restlessness	2,281	58.2	1,638	41.8
**Future concern**				
Employment	1,837	46.9	2,082	53.1
School	3,365	85.9	554	14.1
Financial issues	1,966	50.2	1,953	49.8
Policy	2,437	62.2	1,482	37.8
Mental health	2,654	67.7	1,265	32.3
Stigma	3,860	98.5	59	1.5
Overall health	1,751	44.7	2,168	55.3

Regarding future concerns of the participants, Table 1 shows that the most frequent concern was overall health, with 55.3% of the sample reporting it. Next, with a similar percentage, 53.1% of participants reported feeling concerned about work issues. Similarly, 49.8% of participants reported being concerned about economic issues. In contrast, only 1.5% of participants reported being concerned about stigma.

### Vulnerability Factors Related to Perceived Psychological Impact and Future Concern

#### Age, Perceived Psychological Impact, and Future Concern

When looking at the perceived psychological impact in relation to age, it can be seen in Table 2 that, for all feelings, the average age for people who reported feeling them was significantly lower (*p*'s < 0.01). The widest difference was observed in the feeling of frustration, where those who reported this feeling were on average 35.36 years old, and those who did not report it were on average 43.34 years old (*t* = 21.28, *p* < 0.001). The perceived psychological impact where the least age difference could be observed was worry, where those who reported feeling this way were, on average, 42.24 years old, while those who did not report being worried were 43.77 years old (*t* = 3.10, *p* = 0.002).

**Table 2:** The average age of perceived impact reports on current feelings.

	**Does not report**		**Report**		***t***
	***M***	**DE**	***M***	**DE**	
Fear	43.98	14.38	40.17	13.18	8.24^***^
Concern	43.77	17.77	42.24	13.75	3.10^**^
Frustration	43.34	13.72	35.36	12.50	21.28^***^
Boredom	40.06	13.14	36.51	13.76	21.03^***^
Anxiety	48.03	13.86	39.26	13.16	19.80^***^
Distress	45.59	14.01	38.59	13.19	15.90^***^
Feeling trapped	44.57	13.73	36.85	13.71	14.96^***^
Loss of control	43.30	14.26	37.45	11.24	9.30^***^
Loneliness	43.88	13.58	36.88	15.26	10.76^***^
Sleep problems	44.92	14.17	39.55	13.40	12.05^***^
Inability to relax	44.68	14.05	36.76	12.52	16.49^***^
Loss of freedom	44.21	13.96	39.43	13.89	9.93^***^
Lack of concentration	45.76	13.83	37.57	13.04	18.52^***^
Irritability	45.82	14.42	38.02	12.20	18.18^***^
Restlessness	44.19	13.77	40.72	14.32	7.60^***^

*** *p < 0.001*,

** *p < 0.01*,

* *p < 0.05*.

Comparisons between participants' ages who reported, and did not report, different types of concern during quarantine are shown in Table 3. As a general trend, younger participants reported employment, finances, mental health, stigma, and general health concerns (*p*'s < 0.001). However, no significant differences could be observed in the age of participants that reported school and political concerns.

**Table 3:** Average age of future concern reports.

	**Does not report**		**Report**		***t***
	***M***	***DE***	***M***	***DE***	
Employment	43.73	14.96	41.88	13.25	4.06^***^
School	42.70	14.76	43.05	9.20	−0.76
Financial issues	43.56	14.86	41.94	13.26	3.57^***^
Policy	43.04	13.46	42.26	15.11	1.63
Mental health	45.30	13.88	37.38	13.03	17.31^***^
Stigma	42.84	14.10	36.49	13.30	3.67^***^
Overall health	43.61	13.71	42.04	13.39	3.48^***^

*** *p < 0.001*,

** *p < 0.01*,

* *p < 0.05*.

#### Gender, Perceived Psychological Impact, and Future Concern

Table 4 shows the percentage of men and women who reported different perceived psychological impacts. In general, some statistically significant gender differences can be highlighted. The perception of fear, worry, frustration, anxiety, distress, feeling trapped, loss of control, sleep problems, inability to relax, irritability, and restlessness were reported mostly by women (*p*'s < 0.05). However, 40.1% of the men in the sample reported feeling bored, while only 33.5% of the women reported boredom (χ^2^ = 11.82, gl = 1, *p* < 0.001). In terms of feelings of loneliness, loss of freedom, and lack of concentration, no gender differences were observed.

**Table 4:** Percentage of reporting of perceived impact on current feelings by gender.

	**Male**		**Female**		$\chi_{\left(1\right)}^{2}$
	***F***	**%**	***F***	**%**	
Fear	161	20.5	1,110	35.4	63.37^***^
Concern	480	61.1	2,151	68.7	16.05^***^
Frustration	169	21.5	852	27.2	10.27^**^
Boredom	315	40.1	1,048	33.5	11.82^***^
Anxiety	405	51.5	1,958	62.5	31.13^***^
Distress	213	27.1	1,382	44.1	74.64^***^
Feeling trapped	163	20.7	765	24.4	4.50^*^
Loss of control	52	6.6	321	10.2	9.20^**^
Loneliness	115	14.6	521	16.6	1.70
Sleep problems	239	30.4	1,351	43.1	41.60^***^
Inability to relax	169	21.5	788	25.2	4.39^*^
Loss of freedom	221	28.1	982	31.3	2.93
Lack of concentration	266	33.8	1,177	37.6	3.6
Irritability	2.36	30.0	1,313	41.9	36.6^***^
Restlessness	300	38.2	1,338	42.7	5.13^*^

*** *p < 0.001*,

** *p < 0.01*,

* *p < 0.05*.

*Only the percentages of people who reported having the sensation are included in the table, for ease of reading*.

On gender differences in each of the future concerns studied (see Table 5), more men reported having more future concerns on employment and politics than women (*p*'s < 0.05). However, for mental health and general health concerns, more women reported having them (*p*'s < 0.01). And for school, financial, and stigma concerns, no statistically significant differences were observed.

**Table 5:** Percentage of reporting of future concern by gender.

	**Male**		**Female**		$\chi_{\left(1\right)}^{2}$
	***f***	**%**	***f***	**%**	
Employment	448	57.0	1,634	52.2	5.72^*^
School	101	12.8	453	14.5	1.21
Financial issues	387	49.2	1,566	50.0	0.11
Policy	337	42.9	1,988	36.5	10.43^**^
Mental health	218	27.7	1,047	33.4	9.02^**^
Stigma	11	1.4	48	1.4	0.01
Overall health	371	47.2	1,797	57.4	25.81^***^

*** *p < 0.001*,

** *p < 0.01*,

* *p < 0.05*.

*Only the percentages of people who reported having the concern are included in the table, for ease of reading*.

#### Occupation, Perceived Psychological Impact, and Future Concern

For reporting current perceived psychological impacts and the type of occupation that the participants had, those working as employees were compared with those who were self-employed. The results of this comparison can be seen in Table 6. In general, there was a higher percentage of employed workers who reported feeling bored, anxious, distressed, experiencing sleep problems, an inability to relax, and a lack of concentration (*p*'s < 0.05). For the rest of the perceived psychological impacts, no significant differences were observed.

**Table 6:** Percentage of reporting of impact on current feelings by occupation.

	**Employed worker**		**Self-employed worker**		$\chi_{\left(1\right)}^{2}$
	***f***	**%**	***f***	**%**	
Fear	608	33.6	305	30.1	3.52
Concern	1,227	67.9	682	67.3	0.07
Frustration	410	22.7	228	22.5	0.01
Boredom	565	31.3	280	27.6	3.89^*^
Anxiety	1,135	62.8	544	53.7	21.98^***^
Distress	736	40.7	341	33.7	13.44^***^
Feeling trapped	401	22.2	197	19.4	2.76
Loss of control	179	9.9	88	8.7	0.98
Loneliness	242	13.4	118	11.6	1.62
Sleep problems	740	41.0	354	34.9	9.61^**^
Inability to relax	460	25.5	177	17.5	23.20^***^
Loss of freedom	509	28.2	298	29.5	0.43
Lack of concentration	685	37.9	308	30.4	15.69^***^
Irritability	708	39.2	368	36.3	2.12
Restlessness	715	39.6	416	41.1	0.54

*** *p < 0.001*,

** *p < 0.01*,

* *p < 0.05*.

*Only the percentages of people who reported having the sensation are included in the table, for ease of reading*.

Table 7 shows the results for the type of concern reported by both types of occupation. A majority of self-employed workers reported feeling employment and financial concerns (*p*'s < 0.001). In contrast, a higher percentage of people with dependent employment reported having mental health and general health concerns (*p*'s < 0.001). For school, policy, and stigma concerns, no statistically significant differences were observed between the work mode of the study participants.

**Table 7:** Percentage of reporting of future concern by occupation.

	**Employed worker**		**Self-employed worker**		$\chi_{\left(1\right)}^{2}$
	***f***	**%**	***F***	**%**	
Work	907	50.2	632	62.4	38.45^***^
School	293	16.2	149	14.7	1.00
Economic issues	819	45.3	630	62.2	73.26^***^
Policy	664	36.7	373	36.8	0.01
Mental health	609	33.7	215	21.2	48.26^***^
Stigma	24	1.3	14	1.4	0.01
Overall health	1,039	57.5	487	48.1	22.83^***^

*** *p < 0.001*,

** *p < 0.01*,

* *p < 0.05*.

*Only the percentages of people who reported having the concern are included in the table, for ease of reading*.

### Previous Psychological Support

Table 8 shows the percentage of data on the number of participants who had received pre-quarantine psychological therapy and some particularities of the treatment they received. From all of the sample, 22.3% of participants reported that they had received some type of psychological therapy before quarantine.

**Table 8:** Frequencies and percentages for previous, current, and future psychological support.

	***f***	**%**
**Previous psychological support**		
No	3,045	77.7
Yes	874	22.3
**Therapeutic process progress**		
Few sessions	228	26.1
More than 6	478	54.7
More than 12	168	19.2
**Frequency of psychotherapy sessions**		
More than once a week	29	3.3
Weekly	447	51.1
Less than once a week	79	9.0
Once a month	168	19.2
No fixed frequency	151	17.3
**Reason for consultation**		
Depression	344	39.36
Anxiety	412	47.14
Psychosis	19	2.17
Cognitive and learning problems	17	1.95
Personality problems	52	5.95
Eating disorders	57	6.52
Physical problems	34	3.89
Addictions	23	2.63
Trauma abuse	107	12.24
Grief	122	13.96
Self-esteem	210	24.03
Interpersonal relations	263	30.09
Life and well-being	250	28.60
Work or study	164	18.76
**Current psychological support**		
No	3,620	92.4
Yes	299	7.6
**Experience with current**		
**virtual psychological support**		
Excellent	96	32.1
Odd, but it works	195	65.2
It generates disgust	1	0.3
No good	7	2.3

Of the participants that reported being in treatment, 54.7% reported that they had more than 6 sessions and 19.2% reported that they had more than 12 sessions. In general, the most recurrent frequency was weekly sessions, with 51.1% of the study participants reporting having followed this format. However, 19.2% reported receiving sessions once a month, and 17.3% had no fixed frequency of their sessions.

The most recurrent reason for consultation was anxiety, with 47.14% of participants receiving therapy, followed by depression, with 39.36%. Among the least frequent reasons for consultation in the sample were cognitive and learning problems (1.95%), psychosis (2.17%), and addictions (2.63%). Finally, only 7.6% of participants who had previously been receiving emotional support reported that they were currently attending psychological therapy. When asked about this experience, most of them referred to having a positive experience, or at least feeling that it was helping them (97.3%).

#### Perceived Impact, Previous Psychological Support, and Future Needs

Table 9 shows the associations between the percentage of people who reported having received psychotherapy before quarantine and the feelings they were currently experiencing. In general, people who had received some kind of previous psychotherapy reported feeling most of the perceived psychological impacts studied in higher percentages than those who did not have psychological help (*p*'s < 0.01). However, the association between previous psychological treatment and feeling worried was not statistically significant (χ^2^ = 1.26, gl = 1, *p* = 0.28).

**Table 9:** Percentage of reporting of perceived impact on actual feelings by previous psychological support.

	**No**		**Ye**		$\chi_{\left(1\right)}^{2}$
	***f***	**%**	***F***	**%**	
Fear	926	30.4	345	39.5	25.04^***^
Concern	2,030	66.7	601	68.8	1.26
Frustration	722	23.7	299	34.2	38.16^***^
Boredom	1,012	33.2	351	40.2	14.05^***^
Anxiety	1,736	57.0	627	71.7	60.91^***^
Distress	1,144	37.6	451	51.6	54.82^***^
Feeling trapped	657	21.6	271	31.0	32.89^***^
Loss of control	262	8.6	111	12.7	12.76^***^
Loneliness	429	14.1	207	23.7	45.29^***^
Sleep problems	1,199	39.4	391	44.7	7.87^**^
Inability to relax	678	22.3	279	31.9	33.76^***^
Loss of freedom	893	29.3	310	35.5	11.75^***^
Lack of concentration	1,053	34.6	390	44.6	29.00^***^
Irritability	1,152	37.8	397	45.2	16.05^***^
Restlessness	1,237	40.6	401	45.9	7.5^**^

*** *p < 0.001*,

** *p < 0.01*,

* *p < 0.05*.

*Only the percentages of people who reported having the sensation are included in the table, for ease of reading*.

#### Perceived Impact and the Need for Further Psychological Support

Finishing with the studied perceived psychological impacts, we see in Table 10 the association between the current feelings and reporting the need for psychological support after quarantine. Of the total number of respondents, 43.8% (1,717) reported that they thought they would need some psychological help post-quarantine. These participants reported a statistically significant higher frequency of all the perceived psychological impacts studied than those who reported they would not need help (*p*'s < 0.001).

**Table 10:** Percentage of reporting of perceived psychological impact on current feelings by the need for further support.

	**No**		**Ye**		$\chi_{\left(1\right)}^{2}$
	***f***	**%**	***f***	**%**	
Fear	492	22.3	779	45.4	232.42^***^
Concern	1,351	61.4	1,280	74.5	75.36^***^
Frustration	403	18.3	618	36.0	155.82^***^
Boredom	668	30.3	695	40.5	43.28^***^
Anxiety	1,037	47.1	1,326	77.2	364.68^***^
Distress	611	27.7	984	57.3	348.10^***^
Feeling trapped	389	17.7	539	31.4	99.81^***^
Loss of control	122	5.5	251	14.6	91.27^***^
Loneliness	217	9.9	419	24.4	149.13^***^
Sleep problems	642	29.2	948	55.2	270.60^***^
Inability to relax	329	14.9	628	36.6	243.49^***^
Loss of freedom	560	25.4	643	37.4	65.93^***^
Lack of concentration	600	27.2	843	49.1	197.04^***^
Irritability	681	30.9	868	50.6	154.66^***^
Restlessness	754	34.2	884	51.5	117.21^***^

*** *p < 0.001*,

** *p < 0.01*,

* *p < 0.05*.

*Only the percentages of people who reported having the sensation are included in the table, for ease of reading*.

## Discussion

The goal of the present study was to examine through a survey the perceived psychological impact and future concerns regarding the COVID-19 lockdown in Santiago, Chile. The study also aimed to identify those participants receiving psychological support before quarantine, the psychological effects on them of interruption of the process due to quarantine, and the usefulness of online psychotherapy. It was intended to explore the likely need for psychological support after lockdown.

As we know, worldwide, COVID-19 has caused a parallel epidemic of fear, anxiety, depression, and concern about the future. In this study, being in quarantine for the first 2 weeks of this pandemic had adverse effects on the participants. Mainly, the results show a high presence of general concern and anxiety, consistent with COVID-19 research [e.g., (12, 20, 55)] and past research on the psychological consequences of quarantine during a pandemic (14). Unlike other studies, we did not find a high presence of sleep problems (26), and the results even showed a lower presence of loneliness than was evident in other studies (28).

Regarding future concerns, this study shows how a higher number of participants reported being concerned about their overall health, work, and economic issues. General health has been one of the main concerns during quarantine [e.g., (52)], as the number of deaths has been increasing worldwide, and second outbreaks have even appeared in countries where the pandemic was supposedly under control.

Furthermore, some studies have shown how economic concern is a dimension (38, 39, 56, 57). However, what this study found to be different from the rest is how concerns about work and economic issues had the same relevance as overall health. There are many possible interpretations of this, and some authors [e.g., (53)] have concluded that the perceived psychological impact of COVID-19 has an impact on health concerns, but also quarantine shows other stress-related factors, such as economic or social concerns. On the other hand, South America has been the last continent to be struck by COVID-19, and therefore, the information from the mass media has been intense in terms of health issues, with an increasing number of deaths worldwide. This could explain how health concerns were very prevalent, even though there were fewer deaths than in other countries. The high presence of economic and financial concerns may be understandable because Chile has a particular condition: in October 2019, there was a social outbreak that lasted until the beginning of the pandemic. During this outbreak, 600,000 employees were fired, so the economy was a big concern before COVID-19, and the country was not prepared for this huge possible effect.

When considering vulnerability factors, this study showed that one of the factors is younger people, who reported the most significant perceived psychological impact, with their main issue being frustration. These results are consistent with the findings of Qiu et al. (19) and others (17, 58) of higher emotional distress among individuals aged between 18 and 30. Young people tend to obtain a large amount of information from social media, which can easily trigger stress (45, 59). However, in addition, actual studies on COVID-19 have shown how people aged 60 or above have also reported high levels of psychological distress. This study did not find this result. In fact, older people reported a low presence of perceived psychological impact compared with other ages. This is an unusual result, since every study on COVID-19 and prior pandemics (19) has shown the huge perceived impact on this group. A possible hypothesis is that, as this survey was carried out in the first 2 weeks of quarantine, older people had already been prepared and isolating as a precaution, and therefore, it did not have the same perceived impact as a sudden quarantine, as in other groups, especially the young.

As expected, as in other studies, gender is a vulnerability factor, mainly for women, who reported having more perceived psychological impacts, while men mainly felt bored. We can understand that women had perceived more negative impacts than men, since some of the authors report that women are, in fact, more vulnerable to stress than their counterparts, and they are therefore more susceptible to negative feelings and even post-traumatic stress disorders (12, 19, 31). The fact that the main negative feeling among men was being bored is challenging to understand, but it may have to do with the time of the survey, namely, the first 2 weeks of quarantine, meaning there were still no other perceived impacts on men since it was in the early stages. Women may be more susceptible to connecting to the probable psychological and health impact. These gender differences have been detected in many other studies, for example, Wang et al. (21).

On future concerns, gender differences were also found. Men reported having more concerns about work and politics than women. Meanwhile, women were concerned about their mental and overall health. These differences can probably be explained by traditional male and female gender roles, which are prevalent in South American countries such as Chile. Even though Chile is a developing country, women tend to develop many roles, such as employees, housewives, and childcare providers, while men are usually focused on work and concerned about financially supporting the family (60).

The other group evaluated had not been considered in any other studies, or in studies on COVID-19 or other pandemics. Of those people who were receiving psychological support before quarantine, 22% of the participants had this support before lockdown. Almost half of them had more than six sessions on a weekly basis. Their initial consultations were mainly on anxiety and depression, which coincides with the global prevalence of both disorders worldwide and in Chile (61, 62).

When assessing the perceived psychological impacts that these participants reported, it was found that they had more, and a broader, perceived psychological impact than participants who were not receiving this support before lockdown. These results confirm that the psychological impact of quarantine could more substantially influence people with mental health issues, and, therefore, it may worsen their symptoms because of their high susceptibility to stress compared with the general population (48, 63, 64).

Quarantine disrupts people's lives, especially since there is no possibility of getting around or carrying out daily activities outside the home (11, 12). Therefore, one of the most interrupted activities was the possibility of attending psychological support sessions. For this reason, services are developing expertise in conducting psychiatric assessments and delivering interventions remotely (e.g., by telephone or digitally). There has been worldwide discussion about the change of setting this entails and how therapists are coping with it, but the viewpoint of patients has gone unnoticed. In this study, most of the participants who had virtual support evaluated it as excellent or rare but useful. This is very important because it confirms that these new working practices should be implemented more widely. The results showed that almost half of respondents (43.8%) reported that they believed they needed future psychological support, which confirms and emphasizes the importance of having devices that allow psychological support on a broader scale, benefiting a more significant part of the population. This is highly relevant because the expectation is that, even as contagion decreases worldwide, many people will still be on voluntary quarantine.

This study emphasizes the high presence of psychological effects due to initial quarantine on COVID-19, showing mainly anxiety and concern. As Forte et al. (65) state, this pandemic could even be considered a traumatic event. Vulnerability groups were identified through this study, including women, younger people, and the self-employed, who had a higher presence of perceived psychological effects. Because of its magnitude, this study confirms the need for a national strategic and coordination plan for psychological support, aimed at vulnerable groups, which goes beyond healthcare workers, survivors, or parents in charge of small children.

However, the general population is suffering from negative psychological impacts, such as women, younger people, the self-employed, and those with interrupted psychological help. The delivery of this support must be virtual because of its high potentiality (66, 67), and mainly because this study showed that it is perceived to be effective for patients. Some authors (33, 46) have been developing a specialized psychological intervention for COVID-19 that must be dynamic and flexible enough to adapt quickly to the different phases of the pandemic and the specific groups. Finally, Van Daele et al. (50) have made some specific recommendations for policy-makers on e-mental health and tele-psychotherapy, which must be considered.

### Limitations and Further Research

Because this was an exploratory study, carried out during the first 2 weeks of quarantine in Santiago, it has several limitations. The first relates to the sample, since it is not a probabilistic sample of the Chilean population. Furthermore, the sample is biased because it was obtained through personal contacts and social networks on which we did not explore the participation rate. The sample implied a few sample biases, for example, gender (more women) and level of studies (mostly university or postgraduate). Therefore, the results cannot be generalized to all of the population, but hopefully, they will motivate further studies that cover the Chilean population more generally.

Regarding the survey's validity, our instrument was not a standardized scale designed to measure psychological disorders. So, the results on psychological symptoms during the pandemic are proxies that give us hints about the psychological well-being of the Chilean population. This is especially important because, without a standard survey, some issues with comparability rise. Furthermore, given the metric and heterogeneous nature of our items, reliability measures such as Cronbach's alpha could not be estimated. However, the present research was aimed at screening as many symptoms as possible to offer a descriptive basis for future studies, so we decided to use single items for each symptom based on previous research [e.g., (8, 20, 52, 68)]. Furthermore, our results are in line with reports from the current literature. Nevertheless, it is recommended that future studies use standardized instruments to confirm our findings. Another issue is the limitations self-report assessment has, compared with face-to-face interviews, since the latter may give more and reliable information.

As the pandemic has developed, new research has appeared, showing new variables that must be taken into account for future studies that were not considered for this research. Relevant to this topic is specific symptomatology, since new studies have found a high prevalence of stress, post-traumatic disorder, depression, general anxiety, and a deterioration of sleep quality (25, 26, 53, 65). On the other hand, past adverse experiences must be taken into account since they highly relate to symptomatology. This information is relevant because it may increase psychological vulnerability to COVID (69). Other dimensions must be considered, such as having contact with a family member or friend with COVID-19. Favieri et al. (53) found a low level of psychological well-being among those with such contact.

Moreover, there are groups of people with other vulnerabilities that were not taken into account, such as those suffering from chronic medical conditions, who are more vulnerable to severe disease outcomes (25, 26); health workers on the frontline of COVID-19, who have a higher possibility of being infected; people talking care of children (20); family; and specially survivors (25, 67). In fact, patients who recovered from COVID-19 suffer afterwards from multiple sequelae on several organs and psychiatric symptoms that require a multidisciplinary approach (70).

Finally, many researchers have pointed out [e.g., (52)] that it is essential to understand the potential psychological changes caused by COVID-19 over time. As the pandemic continues, it is expected that the negative impact will have more severe consequences with long-lasting effects (26). One of the study's limitations is that the survey was carried out at one time point. However, Qiu et al. (19) found a decrease in distress levels as time passes. However, a recent study undertaken in the USA, using a longitudinal data set, showed stable levels of stress, anxiety, and depression between two surveys and, therefore, no clinically significant reduction in the perceived psychological impact on the general population (71).

Qiu et al. (19) attributed the decrease of distress to the effective prevention and control measures taken by the Chinese government, which has shown itself to be effective and exerting more control through rapidly closing its borders, increasing traceability, and adequate data information. Chile, on the other hand, started the pandemic with contradictory information and with restrictive measures suddenly adopted. All of this could have provoked an intense perceived psychological impact at the beginning, because of the uncertainty, and as time passes, it may become even more intense with the increase in deaths and the possibility of a second wave.

Either way, a follow-up must be undertaken to identify these patterns and participants' characteristics to develop a target intervention if necessary, for each of the phases of the pandemics.

## Data Availability Statement

The datasets generated for this study are available upon request to the corresponding author.

## Ethics Statement

The studies involving human participants were reviewed and approved by the Institutional Review Board of the Universidad Diego Portales (N°006-2020). The patients/participants provided their written informed consent to participate in this study.

## Author Contributions

PD and VA: conceptualization, methodology, formal analysis, and writing—original draft. KE and SC: writing—reviewing draft and editing. All authors: contributed to the article and approved the submitted version.

## Conflict of Interest

The authors declare that the research was conducted in the absence of any commercial or financial relationships that could be construed as a potential conflict of interest.
